# Partial photoswitching of rod-shaped phycobilisome production in the cyanobacterium *Synechocystis* sp. PCC 6803

**DOI:** 10.1093/pcp/pcaf064

**Published:** 2025-06-11

**Authors:** Mutsumi Kubushiro, Takuto Otsu, Naomi Misawa, Masako Hamada, Toshihiko Eki, Shigeru Kawai, Yuu Hirose

**Affiliations:** Department of Applied Chemistry and Life Science, Toyohashi University of Technology, 1-1 Hibarigaoka, Tempaku, Toyohashi, Aichi 441-8580, Japan; Department of Applied Chemistry and Life Science, Toyohashi University of Technology, 1-1 Hibarigaoka, Tempaku, Toyohashi, Aichi 441-8580, Japan; Department for Advanced Medicine for Viral Infections, National Center for Child Health and Development, National Center for Child Health and Development, 2-10-1 Okura, Setagaya, Tokyo 157-8535, Japan; Department of Applied Chemistry and Life Science, Toyohashi University of Technology, 1-1 Hibarigaoka, Tempaku, Toyohashi, Aichi 441-8580, Japan; Department of Applied Chemistry and Life Science, Toyohashi University of Technology, 1-1 Hibarigaoka, Tempaku, Toyohashi, Aichi 441-8580, Japan; Department of Applied Chemistry and Life Science, Toyohashi University of Technology, 1-1 Hibarigaoka, Tempaku, Toyohashi, Aichi 441-8580, Japan; Department of Applied Chemistry and Life Science, Toyohashi University of Technology, 1-1 Hibarigaoka, Tempaku, Toyohashi, Aichi 441-8580, Japan

**Keywords:** phycobilisome, chromatic acclimation, cyanobacteria

## Abstract

Certain cyanobacteria can alter the structure of their photosynthetic supercomplex phycobilisome (PBS) in response to changes in ambient light color. This process is known as chromatic acclimation (CA) and is classified into seven types (CA1–CA7), each involving distinct modifications of PBS structure. Among them, CA1 is defined as the regulation of the rod-shaped PBS in response to green and red light, maintaining the excitation balance between the two photosystems. *Synechocystis* sp. PCC 6803 (PCC 6803) is a widely used model cyanobacterium for photosynthesis research and harbors the CcaSR photosensory gene cluster for CA1. In this study, we investigated the wavelength dependence of the rod-shaped PBS production in PCC 6803. RNA-Seq analysis revealed that the expression of *cpcL*, which encodes a rod-membrane linker protein of rod-shaped PBS, is upregulated under a wide range of visible light (470–630 nm) and partially suppressed under violet (380–420 nm) and red to far-red light (680–720 nm) conditions. Low-temperature fluorescence emission spectra revealed that the ratio of rod-shaped PBS to hemi-discoidal PBS was highest under green light (530 nm), followed by red (660 nm) and far-red (700 nm) conditions. Furthermore, the isolation of intact PBS fractions using an improved procedure revealed the presence of rod-shaped PBS containing CpcL under these light conditions. The incomplete photoswitching of the rod-shaped PBS production may be due to the adaptation of the photosensory CcaSR system in PCC 6803, which lacks green-absorbing components in its PBSs and provides green-light rich environments in their cell aggregates.

## Introduction

Cyanobacteria are oxygenic phototrophs that utilize solar energy and water as an electron donor to convert CO₂ into organic compounds. Cyanobacteria use a light-harvesting supercomplex called phycobilisome (PBS) to capture and transfer the solar energy to photosystem reaction center complexes, photosystem I (PSI) and photosystem II (PSII) (Grossman et al. 1993, MacColl 1998, Liu et al. 2013, Watanabe and Ikeuchi 2013, Bryant and Canniffe 2018). The overall structure of the PBS exhibits considerable diversity among cyanobacteria. The hemidiscoidal shape of PBS, which consists of a central core and radiating rods, is the most common type in cyanobacteria (Chang et al. 2015, Bryant and Canniffe 2018, Kawakami et al. 2021, Zheng et al. 2021). Bundle-shaped PBS and paddle-shaped PBS are present in the early blanching cyanobacteria (Guglielmi et al. 1981, Jiang et al. 2023). These types of PBS function mainly as antennae of PSII. The rod-shaped PBS, which consists of a single rod, functions as the antenna for PSI and is widely distributed to Cyanobacteria (Marquardt et al. 1997, Kondo et al. 2005, Kondo et al. 2007, Watanabe et al. 2014, Hirose et al. 2019, Shimizu et al. 2023, Zheng et al. 2023). PBSs also exhibit diversity in their absorption properties. Light-harvesting protein in the PBS is called phycobiliprotein and is classified by the absorption maxima as phycocyanin (PC, λ_max_ = 620 nm), phycoerythrin (PE, λ_max_ = 570 nm), phycoerythrocyanin (PEC, λ_max_ = 585 nm), and/or allophycocyanin (APC, λ_max_ = 650 nm) (MacColl 1998). Far-red absorbing APC variant was found in cyanobacteria living under bacteria mats (Li et al. 2016, Ho et al. 2017, Ohkubo and Miyashita 2017, Gisriel et al. 2023). Each phycobiliprotein specifically binds one or more bilin chromophores via covalent linkage to particular cysteine residues (MacColl 1998, Scheer and Zhao 2008, Schluchter et al. 2010, Biswas et al. 2011, Kronfel et al. 2019, Nguyen et al. 2020).

Cyanobacteria can alter the structure and absorption wavelength of PBS in response to ambient light colors via a process called chromatic acclimation (CA) (Kehoe and Gutu 2006, Gutu and Kehoe 2012, Montgomery 2017, Wiltbank and Kehoe 2018). Currently, a total of seven CA types (CA1–CA7) that modify the PBS structure in different ways have been characterized (Sanfilippo et al. 2019, Wang and Chen 2022). In response to green and red light, the amount of rod-shaped PBS (CA1), PE (CA2), both PE and PC (CA3), or PEC (CA7) is regulated. These four CA types are regulated by a cyanobacteriochrome-class photosensor (CcaS or RcaE) that perceives green and red light, whose photosensing mechanism has been elucidated recently at the structural level (Kehoe and Grossman 1996, Hirose et al. 2008, Hirose et al. 2013, Nagae et al. 2021, Nagae et al. 2024). CcaS and its cognate response regulator CcaR (collectively referred to as the CcaSR system) are responsible for regulating CA1, CA2, and CA7, whereas RcaE and its associated response regulators RcaF and RcaC (collectively referred to as the RcaEFC system) are responsible for regulating CA3 (Kehoe and Grossman 1997, Li and Kehoe 2005, Hirose et al. 2010, Sanfilippo et al. 2019, Wang and Chen 2022, Mondal et al. 2024). These four CA types have been distributed to ~15% of phylogenetically diverse cyanobacteria strains (Hirose et al. 2019).

*Synechocystis* sp. PCC 6803 (PCC 6803) is the first photosynthetic organism whose complete genome sequence was determined and widely used as the model organism for research into photosynthesis (Kaneko et al. 1995). PCC 6803 has a hemidiscoidal PBS consisting of a tricylindrical APC core and six PC rods, which are connected via rod-core linker CpcG (Dominguez-Martin et al. 2022, Zheng et al. 2023). PCC 6803 also has a rod-shaped PBS consisting of a PC rod but its basal linker protein is replaced with a rod-membrane linker CpcL (Zheng et al. 2023, Guo et al. 2024). In the genome of PCC 6803, *cpcL* is located within the gene cluster of the CcaSR photosensory system, suggesting that the production of rod-shaped PBS is regulated in response to green and red light (CA1) (Hirose et al. 2008). However, the regulation of the rod-shaped PBS in PCC 6803 has not been characterized in detail. Here, we characterized CA1 in PCC 6803 by RNA-Seq and spectral analysis of the cells and isolated PBS fractions. Our data revealed that the production of rod-shaped PBS in PCC 6803 is not completely switched by green and red light, which may reflect a unique adaptation of the CcaSR system in this cyanobacterium. Our data provide insights into the evolution of the photosensory system for CA in diverse cyanobacteria and contribute to the development of an improved photoswitching system for optogenetic applications.

## Results

### Expression pattern of the rod-membrane linker *cpcL*

The CcaSR system is encoded in a gene cluster consisting of a cyanobacteriochrome-class photosensor *ccaS*, OmpR-class response regulator *ccaR*, and an operon encoding PBS genes. CcaS has an N-terminal transmembrane helix, photosensory domain binding a phycocyanobilin chromophore, and a C-terminal histidine kinase domain (Hirose et al. 2008). CcaS undergoes reversible photoconversion between the green-absorbing form (λ_max_ = 535 nm) and the red-absorbing form (λ_max_ = 672 nm), changing its kinase activity (Hirose et al. 2008, Nagae et al. 2024). Under green light, CcaS phosphorylates CcaR and phosphorylated CcaR binds to the promoter of the PBS operon, inducing their expression (Hirose et al. 2010). Under red light, CcaS dephosphorylates CcaR and suppresses the expression of the PBS operon (Hirose et al. 2010). The PBS operon contains various PBS genes depending on the cyanobacteria strains, including PE in *Geminocystis* sp. NIES-3709, PE and the rod-membrane linker *cpcL* in *Nostoc punctifrome* ATCC 29133, and PEC and *cpcL* in *Leptolyngbya* sp. PCC 6406 (Hirose et al. 2010, Hirose et al. 2017, Hirose et al. 2019).

**Figure 1:**
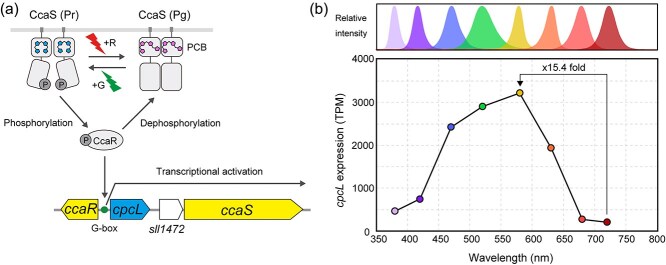
CcaSR gene cluster in PCC 6803 and expression pattern of *cpcL*. (a) Gene organization of CcaSR gene cluster in PCC 6803 and signal transduction pathway. The photoconversion of CcaS between green-absorbing (Pg) and red-absorbing (Pr) states, accompanied by photoisomerization of the phycocyanobilin (PCB) chromophore and a resulting change in its phosphorylation activity, is illustrated. The binding site of CcaR (G-box) is shown accordingly. (b) Expression of *cpcL* was investigated by RNA-Seq. Normalized transcripts per million (TPM) values for each light color condition are shown, along with the emission spectra of the illuminated LEDs.

In PCC 6803, the CcaSR cluster contains *cpcL* (Fig. 1a), suggesting that this strain regulates the production of the rod-shaped PBS by green and red light. The CcaSR gene cluster also contains the *sll1472* gene, which encodes a small protein of unknown function that shares structural similarity with response regulators (Varadi et al. 2022). We first investigated the expression of *cpcL* under green and red light in PCC 6803 and found that the *cpcL* expression was not completely suppressed under red light (data not shown). Therefore, we further investigated the expression pattern of *cpcL* by RNA-Seq in PCC 6803 under different light conditions spanning the full spectrum, including ultraviolet (peaking at 380 nm), violet (420 nm), blue (470 nm), green (520 nm), yellow (580 nm), orange-red (630 nm), deep red (680 nm), and far-red (720 nm). Notably, the highest expression of *cpcL* was observed under yellow light conditions (Fig. 1b). Compared to yellow light conditions set at 100%, its expression was 90.3% under green, 75.3% under blue, and 60.3% under orange-red conditions (Fig. 1b), suggesting that *cpcL* is expressed across a wide range of visible light wavelength. The expression of *cpcL* decreased to 23.2% under violet, 14.5% under ultraviolet, 8.5% under deep-red, and 6.5% under far-red light conditions (Fig. 1b). The ON/OFF dynamics of the *cpcL* expression was highest, reaching 15.4-fold, under yellow versus far-red light conditions.

### Purification of the rod-shaped and hemidiscoidal PBSs

**Figure 2:**
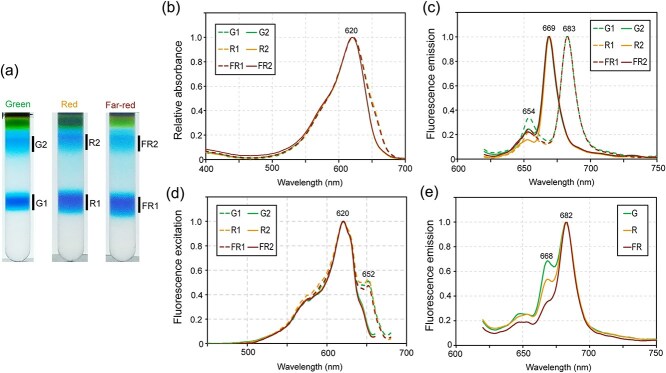
Isolation of rod-shaped and hemidiscoidal PBSs. (a) Fractionation of the PBSs on the sucrose density gradient. (b–d) Absorption spectra (b), low-temperature fluorescence emission spectra (c), and low-temperature fluorescence excitation spectra (d) for each PBS fraction. The excitation wavelength was 610 nm for all PBS fractions in (b). The emission wavelength was 690 nm for G1, R1, and FR1 fractions and 670 nm for G2, R2, and FR2 fractions. (e) Low-temperature fluorescence emission spectra of the cell homogenates before ultracentrifugation on the sucrose density gradient with 610 nm excitation. Spectra were normalized to the emission from APC core of the hemidiscoidal PBS.

To investigate the regulation of the rod-shaped PBS containing CpcL at the protein level, we purified PBSs from PCC 6803 cells, which were cultured under green (530 nm, fluorescent lamp), red (660 nm, fluorescent lamp), and far-red (700 nm, LED) light conditions. Previously, PBS of PCC 6803 was purified using conventional aqueous two-phase separation using Triton X-100 in high phosphate buffer, yielding smear bands showing fluorescence emission characteristic of the rod-shaped PBS (Kondo et al. 2005). CpcL has a hydrophobic transmembrane helix in its C-terminus and preferentially transferred to the Triton layer (Kondo et al. 2007, Hirose et al. 2019). We identified that the supplement of *n*-dodecyl-β-*D*-maltoside (DDM) effectively suppressed the phase separation of Triton X-100 and prevented aggregation of the PBSs during ultracentrifugation on the sucrose density gradient (Hirose et al. 2019). With this improvement, PBS fractions of PCC 6803 were successfully separated into two prominent blue bands on the sucrose density gradient (Fig. 2a). The lower (band 1) and upper (band 2) bands from green (G), red (R), and far-red (FR) acclimated cells were designated as combinations (e.g. G1 for the lower band from green-acclimated cells).

Absorption spectra of all fractions (G1, G2, R1, R2, FR1, and FR2) had a PC peak at 620 nm (Fig. 2b). G1, R1, and FR1 fractions exhibited a shoulder peak of APC at 650 nm, whereas G2, R2, and FR2 fractions did not (Fig. 2b). Low-temperature fluorescence emission spectra showed that G1, R1, and FR1 fractions exhibited emission from APC core peaking at 680 nm, whereas G2, R2, and FR2 fractions showed emission from the rod-shaped PBS peaking at 669 nm (Fig. 2c) (Watanabe and Ikeuchi 2013, Hirose et al. 2019, Guo et al. 2024). Low-temperature fluorescence excitation spectra confirmed the absence of APC peaking at 652 nm in the G2, R2, and FR2 fractions (Fig. 2d). These results demonstrated that the presence of the hemidiscoidal PBS (band1) and rod-shaped PBS (band2) in PCC 6803 acclimated to green, red, and far-red light conditions. To estimate the ratio of the rod-shaped PBS to hemidiscoidal PBS, we measured low-temperature fluorescence emission spectra of the cell homogenates before the ultracentrifugation on the sucrose density gradient (Supplementary Fig.  S1). The obtained spectra were fitted with the emission spectra of the purified PBS fractions (Supplementary Fig.  S2) (Watanabe and Ikeuchi 2013, Hirose et al. 2019). When the 683 nm peak of hemidiscoidal PBS was set to ~100%, the 669 nm peak of rod-shaped PBS was ~65% under green light, ~45% under red light, and ~25% under far-red light conditions (Fig. 2e and Supplementary Fig.  S2). Thus, the ratio of rod-shaped PBS to hemidiscoidal PBS increased under green light conditions, with a relative ON/OFF dynamic of ~2.6-fold between green and far-red light at the protein level.

### SDS-PAGE and LC–MS/MS assignment

**Figure 3:**
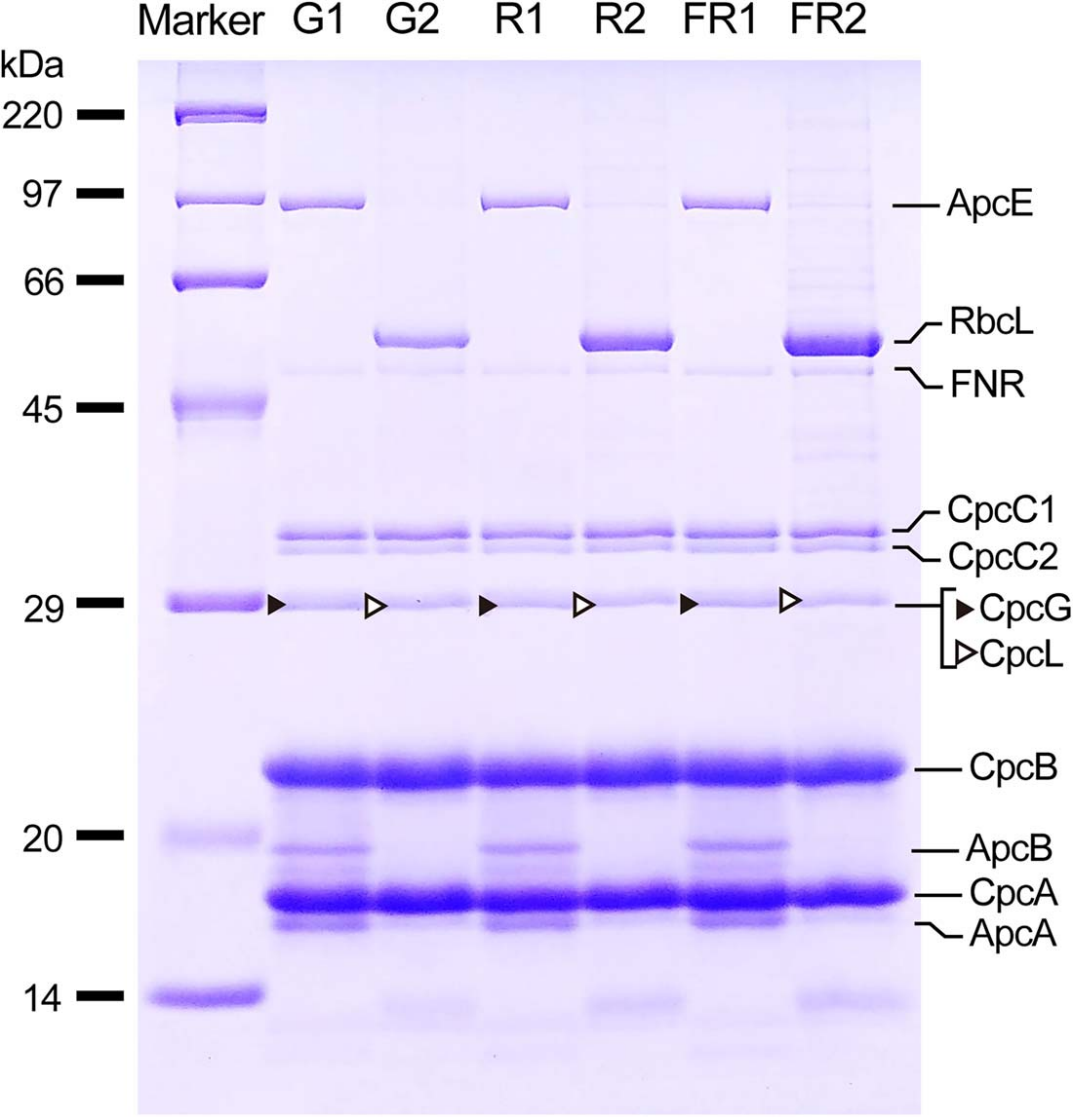
SDS-PAGE and band assignment. The PBS fractions from the sucrose density gradient were separated by SDS-PAGE and stained with CBB. A band corresponding to CpcG/CpcL was assigned by LC–MS/MS (Supplementary Table S1). Other bands of PBS were assigned based on previous N-terminal peptide sequencing (Kondo et al. 2005). RbcL was assigned according to previous LC–MS/MS analysis (Otsu et al. 2022).

The peptide composition of the isolated PBS fractions was analyzed by sodium dodecyl sulfate polyacrylamide gel electrophoresis (SDS-PAGE). Terminal emitter of the APC core of the hemidiscoidal PBS (ApcE, M.W. of apoprotein 100.3 kDa), ApcA (17.4 kDa), ApcB (17.2 kDa) is present in G1, R1, and FR1 fractions (Fig. 3). CpcA (17.6 kDa), CpcB (18.1 kDa), CpcC1 (32.5 kDa), CpcC2 (30.8 kDa), Ferredoxin:NADP^+^ reductase (FNR, 46.4 kDa) is present in all fractions, indicating that these components are common for the rod-shaped and hemidiscoidal PBSs. The FNR band is of lower intensity than that of other PBS proteins and shows similar intensity across all fractions (Fig. 3), suggesting that FNR is associated with only a subset of rods and is present at similar proportions in rod-shaped and hemidiscoidal PBSs. A major band, which corresponds to RuBisCO large chain (RbcL, 52.5 kDa), is present in G2, R2, and FR2 fractions (Fig. 3). A previous Western blot analysis in PCC 6803 proposed that CpcL (28.5 kDa) has different mobility in SDS-PAGE compared to CpcG (M.W. 28.9 kDa), the rod-core linker protein for the hemidiscoidal PBS (Kondo et al. 2005). However, we observed no difference in the three PBS linker polypeptide bands corresponding to CpcC1, CpcC2, and CpcG in SDS-PAGE between the rod-shaped and hemidiscoidal PBS fractions (Fig. 3). Therefore, we analyzed the peptide composition of the band corresponding to CpcG in the previous study by Liquid chromatography-tandem mass spectrometry LC–MS/MS. CpcG was identified in the G1, R1, and FR1 fractions with the highest Mascot Score, the greatest number of detected peptides, and the highest sequence coverage (Supplementary Table S1). In contrast, CpcL was identified in the G2, R2, and FR2 fractions highest values for these parameters (Supplementary Table S1). A small amount of CpcG fragment was detected in the G2, R2, and FR2 fractions, suggesting that partially dissociated PC rods from the hemidiscoidal PBS is present in these fractions. Low-temperature fluorescence emission spectra suggested that the dissociated PC rod, with an emission peak around 654 nm, was not a major component (Fig. 2c). These data demonstrate that CpcG and CpcL are associated with hemidiscoidal PBS and rod-shaped PBS fractions, respectively, and that both proteins exhibit the same mobility in SDS-PAGE.

### Spectroscopic analysis *in vivo*

**Figure 4:**
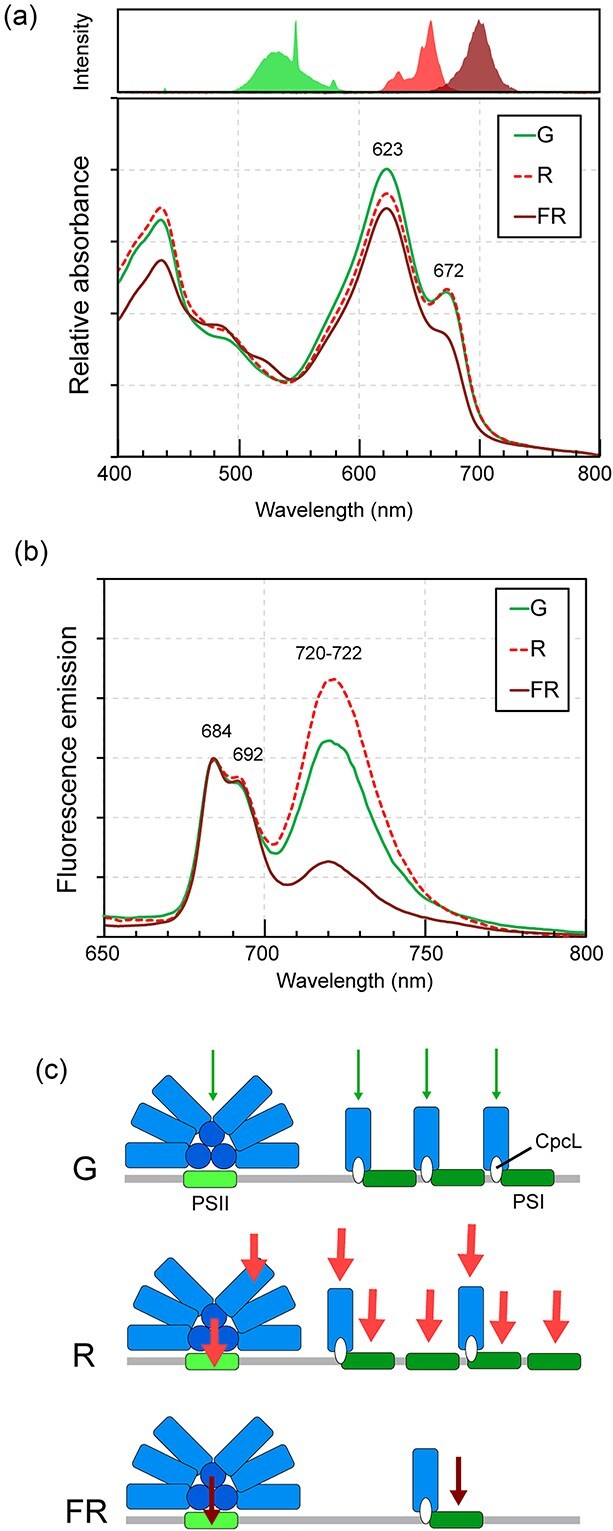
Spectroscopic analysis of cells. (a) Absorption spectra of PCC 6803 cells acclimated to green (G), red (R), and far-red (FR) light conditions. Spectra were normalized at OD_750_. The emission spectra of the illuminated LED (FR) and fluorescence lamps (G and R) are shown accordingly. (b) Low-temperature fluorescence emission spectra of the acclimated cells with 440 nm excitation. Spectra were normalized by emission from PSII at 684 nm. (c) A hypothetical model illustrating changes in the amounts of rod-shaped PBS, hemidiscoidal PBS, PSII, and PSI in PCC 6803. For explanatory purposes, the amounts of PSII and hemidiscoidal PBS are shown as equal between all light conditions.

To investigate the effects of different light qualities on PCC 6803, cells were fully acclimated to green, red, and far-red light and then analyzed using absorption and low-temperature fluorescence spectroscopy (Fig. 4a and b). In cyanobacteria, the PSI:PSII stoichiometry changes from ~1:1 to 4:1 in response to light wavelength (Fujita and Murakami 1987). This shift is primarily attributed to changes in PSI levels, while PSII levels remain relatively constant. The chlorophyll *a* content of the PSI monomer is ~3-fold higher than that of the PSII monomer (Jordan et al. 2001, Umena et al. 2011). Taken these points together, the relative abundance of hemidiscoidal PBS, rod-shaped PBS, PSII, and PSI were shown in a model (Fig. 4c). The excitation of these photosynthetic apparatus by green, red, and far-red light is shown as arrows (Fig. 4c).

Cellular absorption spectra normalized at OD_730_ showed that the amount of PC peaking at 623 nm is higher under green compared to red light conditions, while the amount of chlorophyll *a* peaking at 672 nm unchanged between the two light conditions (Fig. 4a). Low-temperature emission spectra showed that the ratio of PSI emission (720–722 nm) to PSII emission peaks (684 and 692 nm) was lower under green than under red light conditions (Fig. 4b), which is consistent with a recent study on comprehensive light color acclimation in PCC 6803 (Zavřel et al. 2024). Considering the ratio of rod-shaped PBS to hemidiscoidal PBS increased under green light than under red light conditions (Fig. 2e), these results suggest that PCC 6803 enhances the formation of rod-shaped PBS-PSI supercomplex to maintain the excitation of PSI under green light (Fig. 4c). On the other hand, the amounts of PC and chlorophyll *a* were lower under far-red light than under green and red light conditions (Fig. 4a). Low-temperature emission spectra showed a decrease in PSI/PSII ratio under far-red light than under green and red light conditions (Fig. 4b). Considering the ratio of rod-shaped PBS to hemidiscoidal PBS decreased under far-red light condition (Fig. 2e), PCC 6803 reduces the formation of rod-shaped PBS–PSI supercomplex under far-red light (Fig. 4c), which preferentially excites the chlorophyll *a*-rich PSI. These results suggest that the formation of rod-shaped PBS–PSI supercomplex is regulated in concert with the PSI/PSII ratio to ensure balanced excitation of the two photosystems during CA1.

## Discussion

In this study, we characterized the regulation of the rod-shaped PBS (CA1) in PCC 6803, the most widely used cyanobacterial strain in photosynthesis research. The expression of *cpcL* was upregulated under blue to orange-red light (470–630 nm) and partially suppressed under violet (380–420 nm) and deep red to far-red light (680–720 nm) conditions. The ON/OFF dynamics of the rod-shaped PBS production were modest at both transcription and protein levels compared with other CA-performing cyanobacteria strains. Isolation of the intact PBS fractions using an improved procedure and LC–MS/MS assignment confirmed the presence of rod-shaped PBS containing CpcL. To our knowledge, this is the first comprehensive study that analyzes CA1 responses at the transcriptional, protein, and cellular levels in the cyanobacteria strains having only PC for the rod component of the PBS.

**Figure 5:**
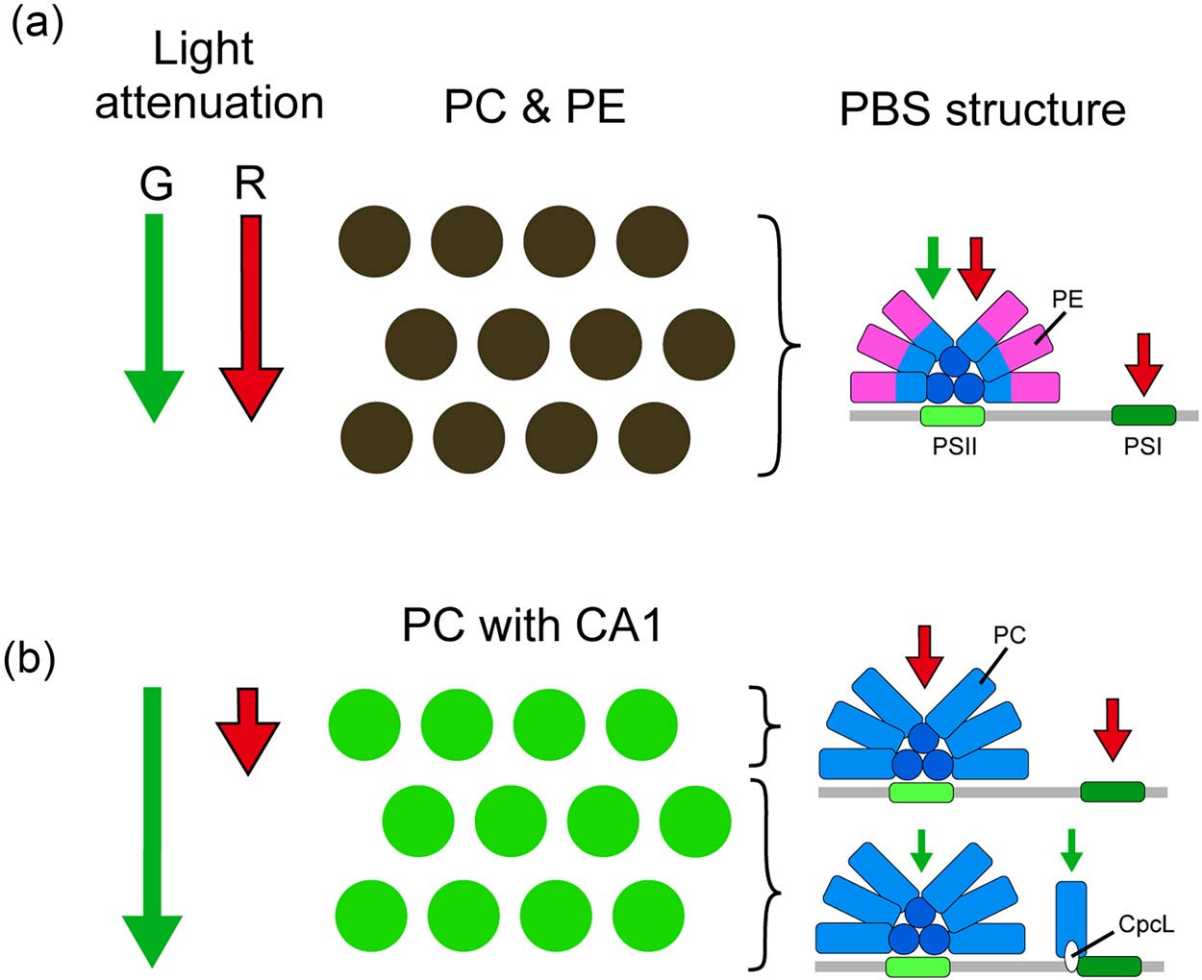
A hypothetical model of CA in cell aggregates. The cyanobacterial mats of PE/PC-containing strain (a) and PC-containing CA1 strain (b) are shown. The structure of PBSs (right) and attenuation of green and red light (left) are also shown.

We previously showed that CA systems are preferentially distributed in cyanobacteria strains of Sections II–V (Hirose et al. 2019). These cyanobacteria are often exposed to green-light enriched conditions by self-shading of red light in natural environments, such as cell aggregates and bacterial mats. PCC 6803 is a unicellular cyanobacterium but can produce exopolysaccharides and form cell aggregates (Maeda et al. 2021). Therefore, we speculate that CA1 functions as a shade sensor in PCC 6803 to acclimate changing light conditions within the aggregates, maximizing its photosynthesis efficiency. To understand the physiological role of CA1 in PCC 6803, let us consider cell aggregates of a unicellular cyanobacterial strain that harbors hemidiscoidal PBS consisting of PE and PC. In this strain, PSII is excited by green and red light through hemidiscoidal PBS, whereas PSI is excited by red light through its own chlorophylls (Fig. 5a). The excitation balance of PSII and PSI are maintained throughout the layers as green and red light equally attenuates in the layers (Fig. 5a). On the other hand, if the strain harbor only PC, like PCC 6803, red light is absorbed and efficiently attenuated in the surface layer, whereas unabsorbed green light can penetrate deeper layers (Fig. 5b). Such a green-light rich environment is present in 0.1–1.0 mm surface of the bacterial mat consisting of PC-containing *Thermosynechococcus* (Martinez et al. 2019). In the deeper layers, PSII and PSI are excited by green light through hemidiscoidal and rod-shaped PBSs, respectively, although their excitation is not so efficient due to the limited absorption of green light with PC (Fig. 5b). Thus, the presence of rod-shaped PBS under the green-light rich environment effectively maintains the excitation balance of PSII and PSI.

We demonstrated that the ON/OFF dynamics of *cpcL* expression in PCC 6803 exhibit a 15.4-fold change (Fig. 1b). This level of regulation is relatively modest compared to the fold changes observed under green versus red light in other CA strains containing PE or PEC, such as *Geminocystis* sp. NIES-3709 (177-fold), *Geminocystis* sp. NIES-3708 (97-fold), *Pleurocapsa* sp. PCC 7319 (429-fold), and *Leptolyngbya* sp. PCC 6406 (1694-fold) (Hirose et al. 2010, Hirose et al. 2017, Hirose et al. 2019, Otsu et al. 2022). We also demonstrated that *cpcL* expression was upregulated under blue (420 nm) to orange-red (630 nm) light conditions in PCC 6803, covering most of the visible spectrum (Fig. 1b). This result is consistent with a previous DNA microarray study in PCC 6803, which reported that *cpcL* is expressed under blue (450 nm), orange-red (625 nm), and red light (660 nm) conditions (Luimstra et al. 2020). At the protein level, we demonstrated that the relative ON/OFF dynamics of rod-shaped PBS to hemidiscoidal PBS is ~2.6-fold under green versus far-red conditions (Fig. 2e). We also confirmed the presence of a prominent rod-shaped PBS band in the sucrose density gradient under green, red, and far-red light conditions (Fig. 2a). Consistently, a previous Western blot study in PCC 6803 showed that CpcL protein, fused with a reporter tag, increased by only 2- to 4-folds under green light (520 nm) compared with under red light (660 nm) (Gao et al. 2018). Thus, the chromatic regulation of rod-shaped PBS consisting of CpcL is moderate in PCC 6803 compared to other CA strains, both at transcription and protein levels. Why does PCC 6803 strain employ such a low-performance photoswitching system for CA1? Our model suggests that the cell aggregate of PC-containing strain is enriched with green light compared with those of PE/PC-containing or PEC/PC-containing strains (Fig. 5). This would lead to the activation state of the CcaSR system persisting in the ON state over a broader range in the aggregate (Fig. 5b). Therefore, we speculate that the photoswitching of the CcaSR system was biased toward the ON state in PCC 6803, resulting in a leaky OFF state during the evolutionary adaptation to natural environments. Analyzing the switching activity of the CcaSR system in other CA1 strains that contain only PC will provide further validation of our hypothesis.

The CcaSR system has been used for optogenetic control of gene expression in various organisms (Lindner and Diepold 2022). Incomplete photoswitching activity of the CcaSR system has also been observed in *Escherichia coli*, and efforts have been made to improve its switching dynamics (Ong and Tabor 2018). The photoconversion of CcaS from PCC 6803 is fully reversible (Hirose et al. 2008), suggesting that the incomplete ON/OFF switching is not due to the inefficient green/red photoconversion of the CcaS protein. Knockout experiments of *ccaS* or *ccaR* gene in *Nostoc punctiforme* ATCC 29133 suggested that CcaS has both kinase activity under green light and diphosphatase activity under red light, and CcaR is the sole activator of the target PBS operon (Fig. 1a) (Hirose et al. 2010). These points suggest that the activation of the phosphorylation cascade of the CcaSR system is responsible for the incomplete ON/OFF switching activity in PCC 6803. We speculate that balance of the kinase/phosphatase activity of CcaS and/or imbalance of the amount of CcaS-to-CcaR would occur during the adaption of the CcaSR system in PCC 6803 (Fig. 1a) (Schmidl et al. 2014). Our data emphasize the importance of choosing the appropriate host cyanobacterial strains to utilize their photoswitching system for optogenetic applications. Transcriptional analysis of PCC 6803 to different environmentally relevant stimuli revealed that the transcripts encoding *cpcL*, *sll1472*, and *ccaS* were downregulated under conditions with heat stress, CO_2_ limitation, dark, high light, iron limitation, and nitrogen limitation (Kopf et al. 2014). It is also important to understand the signal transduction network of global transcriptional regulation and its involvement with specific regulation of PBS genes by light colors in cyanobacteria.

## Materials and Methods

### Strain and growth conditions

There are several laboratory strains of PCC 6803, each carrying distinct mutations on their genome (Kanesaki et al. 2012). Because the *ccaS* photoreceptor gene is disrupted by an insertional transposon in the sequenced GT-Kazusa strain (Okamoto et al. 1999), we used a substrain of the PCC strain that retains the *ccaS* gene and exhibits positive phototaxis (referred to as PCC-P) (Yoshihara et al. 2001, Kanesaki et al. 2012). PCC 6803 was cultivated in a 50 ml liquid BG11 medium buffered with 20 mM HEPES-NaOH pH 7.8 in a glass test tube (Φ30 mm) bubbled with air containing 1% (v/v) CO_2_ at 26°C under continuous light (Rippka et al. 1979). Cultures were illuminated continuously with LED light sources of different wavelengths, and the photon flux density was adjusted to 20 μE m^−2^ s^−1^ for each condition. Emission spectra of fluorescent lamps and light-emitting diodes (LEDs) were shown (Figs 1b and 4a). For RNA-Seq, the cells were grown under white light and then acclimated to each light condition for 24 h. For PBS isolation and cell spectroscopy, the cells were fully acclimated to green, red, or far-red light conditions through more than three rounds of subculturing under each light condition. These analyses were conducted without technical replication.

### Spectroscopy

Absorption spectroscopy and low-temperature fluorescence spectroscopy at 77 K were performed as previously described (Hirose et al. 2017). The concentration of chlorophyll *a* (micrograms Chl *a* per milliliter) was calculated as previously described (Meeks and Castenholz 1971). Cells were diluted to 5 μg Chl *a*/ml and were pre-incubated in the dark for 5 min before the measurement of chlorophyll fluorescence at 77 K.

### RNA sequencing (RNA-Seq) analysis

Extraction of RNA and library preparation were performed as previously described (Hirose et al. 2017). The single end of the libraries was sequenced on the MiSeq sequence with the MiSeq Reagent kit v3 (150 cycles; Illumina). 1.34–2.15M reads were mapped to the reference genome of PCC 6803 (GCA_000009725.1). Mapping, read count, and normalization were performed as previously described (Otsu et al. 2022). The expression level of *cpcL* was specifically extracted from the transcriptome data and presented as TPM.

### Isolation of PBS

Purification of PBS was performed at room temperature. PCC 6803 cells were harvested by centrifugation at 13 000 × *g* for 5 min and resuspended in 0.8 M potassium phosphate buffer pH 7.0. The collected cells were resuspended with 500 μl 0.8 M potassium phosphate pH 7.0 and disrupted with zirconia/silica beads (ϕ0.1 mm, BioSpec) on TissueLyser II (Qiagen) for 3 min at 30 Hz. The homogenates were supplemented with 2.0% (v/v) Triton X-100 and 1.0% (w/v) DDM, then incubated for 30 min in the dark with a gentle rotation. The cellular lysate was centrifuged at 21 600 × *g* for 20 min. The supernatant (550 μl) was loaded onto a 7.5%–30% (w/v) linear sucrose density gradient containing 0.80 M phosphate buffer containing 0.2% (v/v) Triton and 0.2% (w/v) DDM (Beckman Coulter Brea, CA). The gradient was centrifuged at 288 000 × *g* for 5 h at 20°C in an SW41Ti rotor (Beckman Coulter). The rest of the supernatant was diluted with potassium phosphate buffer and subjected to low-temperature fluorescence spectroscopy to estimate the ratio of the rod-shaped PBS to hemidiscoidal PBS as described below. Each colored fraction was collected from the top of the sucrose gradient using a micro syringe equipped with an L-shaped needle. To remove the sucrose, the PBS fractions were precipitated by increasing the potassium phosphate concentration to 1.5 M and centrifugation at 21 600 × *g* for 20 min. The precipitated PBS was washed with the same buffer and then resuspended in the 0.80 M phosphate buffer containing 0.1% (w/v) DDM. Each fraction was subjected to absorption and low-temperature fluorescence spectroscopy. The fluorescence emission spectra of the supernatant samples (G, R, and FR) were simulated by a combination of the spectra of rod-shaped (R2 fraction) and hemi-discoidal (R1 fraction) PBSs at specific ratios (Supplementary Fig.   S2).

### SDS-PAGE and LC--MS/MS analyses

Purified PBS fractions were incubated with 10% (v/v) trichloroacetic acid (TCA) for 10 min on ice and precipitated by centrifugation at 21 600 × *g* for 10 min at 4°C. The colored pellets were washed once with 10% (v/v) of TCA and twice with acetone and were then resuspended in AE-1430 EzApply (ATTO, Tokyo, Japan). The samples were incubated at 95°C for 5 min, separated on a 15% acrylamide gel, and then stained with Coomassie Brilliant Blue R-250 (CBB). The stained bands were cut out using disposable razors and subjected to in-gel trypsin digestion and LC–MS/MS analysis as reported previously (Otsu et al. 2002).

## Supplementary Material

Fig_S1_pcaf064

Fig_S2_pcaf064

Table_S1_pcaf064

Supplementary_materials_pcaf064

## Data Availability

Gene expression data of the CA1 cluster of *Synechocystis* sp. PCC 6803 under irradiation at different light wavelengths are provided in Supplementary Table S2. For each light condition, TPM values were obtained from a single biological sample (*n* = 1).
