# Supplementary figures and images for: Partial photoswitching of rod-shaped phycobilisome production in the cyanobacterium Synechocystis sp. PCC 6803
Source: Plant Cell Physiol. 2025 Jun 11;66(9):1274–83. doi: 10.1093/pcp/pcaf064 (PMC12461855; doi:10.1093/pcp/pcaf064)

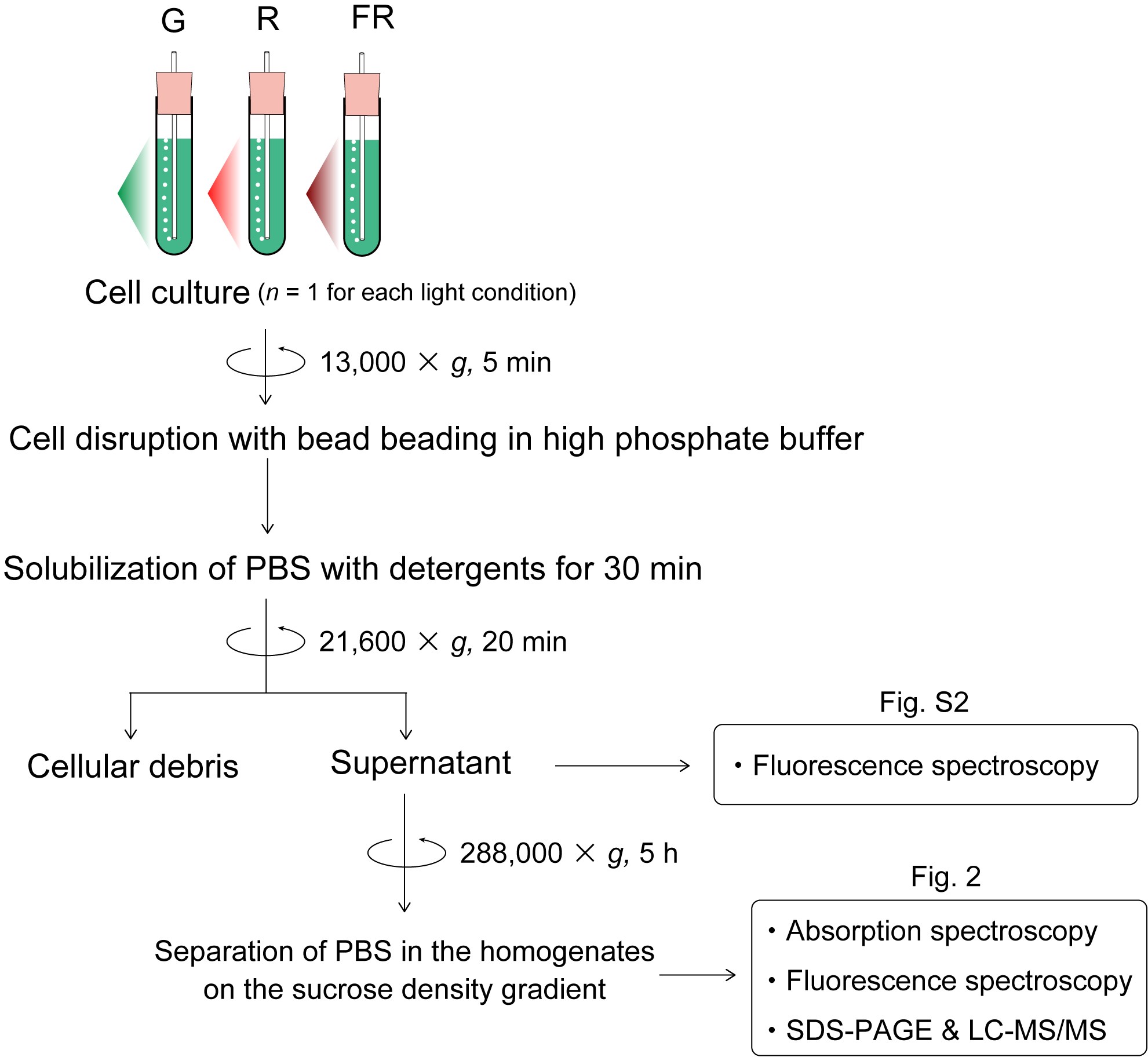

Supplement: Fig_S1_pcaf064 [file fig_s1_pcaf064.jpeg]

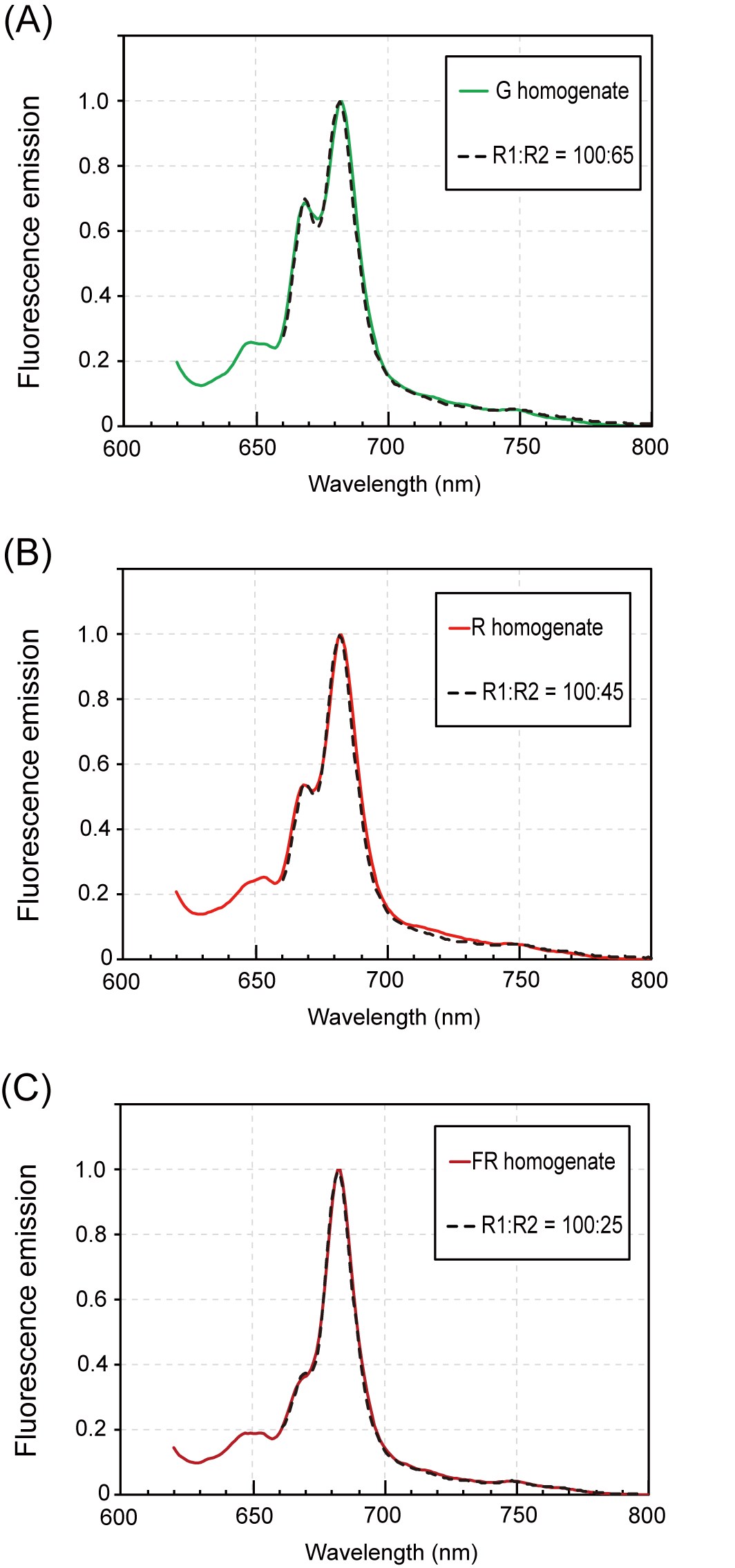

Supplement: Fig_S2_pcaf064 [file fig_s2_pcaf064.jpeg]
