# Supplementary material for: Partial photoswitching of rod-shaped phycobilisome production in the cyanobacterium Synechocystis sp. PCC 6803
Source: Plant Cell Physiol. 2025 Jun 11;66(9):1274–83. doi: 10.1093/pcp/pcaf064 (PMC12461855; doi:10.1093/pcp/pcaf064)
Supplement: Supplementary_materials_pcaf064 [file supplementary_materials_pcaf064.docx]

**Supplementary materials**

**Figure S1. Outline of the purification procedure for PBS**

**Figure S2. Estimation of the ratio of rod-shaped PBS to hemidiscoidal PBS**

The low-temperature fluorescence emission spectra of the cell homogenates in Fig. 2E were fitted using those of the R1 and R2 fractions in Fig. 2C at the ratios shown.

**Table S1. LC-MS/MS analysis of the band corresponding to CpcG/CpcL**
